# Activation gating in HCN2 channels

**DOI:** 10.1371/journal.pcbi.1006045

**Published:** 2018-03-22

**Authors:** Sabine Hummert, Susanne Thon, Thomas Eick, Ralf Schmauder, Eckhard Schulz, Klaus Benndorf

**Affiliations:** 1 Institut für Physiologie II, Universitätsklinikum Jena, Friedrich-Schiller-Universität Jena, Jena, Germany; 2 Fachhochschule Schmalkalden, Fakultät Elektrotechnik, Blechhammer, Schmalkalden, Germany; University of California San Diego, UNITED STATES

## Abstract

Hyperpolarization-activated cyclic nucleotide-modulated (HCN) channels control electrical rhythmicity in specialized brain and heart cells. We quantitatively analysed voltage-dependent activation of homotetrameric HCN2 channels and its modulation by the second messenger cAMP using global fits of hidden Markovian models to complex experimental data. We show that voltage-dependent activation is essentially governed by two separable voltage-dependent steps followed by voltage-independent opening of the pore. According to this model analysis, the binding of cAMP to the channels exerts multiple effects on the voltage-dependent gating: It stabilizes the open pore, reduces the total gating charge from ~8 to ~5, makes an additional closed state outside the activation pathway accessible and strongly accelerates the ON-gating but not the OFF-gating. Furthermore, the open channel has a much slower computed OFF-gating current than the closed channel, in both the absence and presence of cAMP. Together, these results provide detailed new insight into the voltage- and cAMP-induced activation gating of HCN channels.

## Author summary

Hyperpolarization-activated cyclic nucleotide-modulated (HCN) channels are tetrameric voltage-controlled ion channels in the cell membrane of specialized nerve and heart cells. Their main function is to generate a so-called pacemaker current which plays a key role in the generation of electrical rhythmicity. A special messenger molecule, cAMP, synthesized within these cells at sympathetic stimulation, can bind to these channels, thereby enhancing channel opening evoked by voltage. The mechanism of this dual activation is still poorly understood. Here we quantified this duality of activation for HCN2 channels by globally fitting hidden Markovian state models to extensive sets of data. We propose that activation of this tetrameric channel requires for a full description only two voltage-dependent steps that are followed by a voltage-independent opening step of the channel pore. According to this model analysis cAMP exerts multiple effects on channel activation: It notably accelerates the charge movement of the voltage-dependent steps and reduces the number of the involved electrical charges. Furthermore, it introduces an additional closed state and stabilizes the open pore. Together, our results provide new insight into the duality of voltage- and cAMP-induced activation of HCN channels.

## Introduction

Hyperpolarization-activated cyclic nucleotide-modulated (HCN) [1–3] ion channels generate electrical rhythmicity in both specialized neuron [4–11] and cardiomyocytes [12–15] by evoking the pacemaker current *I*_h_ (*I*_f_, *I*_q_). The primary activating stimulus for HCN channels is hyperpolarization of the plasma membrane [16, 17], as proceeding in the repolarization phase of the action potential. In addition to this primary stimulus, sympathetic activity enhances the activation of HCN channels by the second messenger cAMP, binding to the channels [18–22]. The resulting increase of the pacemaker current by cAMP binding evokes an acceleration of electrical rhythmicity.

Evolutionary relationships show that HCN channels belong to the superfamily of tetrameric voltage-gated ion channels [23] in which each subunit contains in the transmembrane part a voltage sensor domain (VSD) and a pore domain. In HCN channels, a cyclic nucleotide-binding domain (CNBD) is additionally incorporated in the C-terminus [22]. In mammals four related genes have been identified to encode for the subunits HCN1 to HCN4 [17, 24]. Each of these subunits can form functional homotetrameric channels [5, 25, 26], but also functional heterotetrameric channels have been reported [27–30].

The recent publication of cryo-electron microscopy structures of the HCN1 isoform at 3.5 Å resolution both in the absence and presence of cAMP [31] is a milestone on the way to further understand the work of HCN channels. The authors provide for the first time a structurally-based scenario for the duality of voltage- and cAMP-induced activation and the unusual reversed polarity of activation within the superfamily of voltage-gated channels. In the setting of a depolarized voltage sensor, three effects are supposed to stabilize the gate in a closed position: an unusually long S4 helix touching the C-linker, a special packing arrangement of the S4, S5 and S6 helices and a particular 3-α-helical HCN domain preceding S1. A hyperpolarization-driven downward motion of the S4 helix would disrupt these stabilizing effects and movement of the S6 helices would open the gate. The activating effect of cAMP-binding to the CNBD is explained by evoking a concerted rotation of cytoplasmic domains enhancing opening of the gate by promoting this disruption of stabilizing effects. A similar mechanism for the action of cyclic nucleotides, a relief of auto-inhibition, has already been proposed earlier [32]. Notably is also that the structure of the CNBD as part of whole channel is closely similar to that determined previously for isolated CNBDs of three mammalian HCN isoforms [33–35].

Concerning the kinetics of voltage-gated activation, DiFrancesco [18] used a Hodgkin-Huxley type model [36] to describe native HCN channel kinetics, proposing that upon channel activation two subunits gate. In subsequent analyses on three HCN isoforms, a cyclic allosteric Monod-Wyman-Changeux (MWC) type model [37] with five closed and five open states was used to interpret experimental data, containing four independently operating voltage sensors and five allosteric closed-open isomerizations [38]. This approach allowed the authors to estimate several rate constants in the model, suggesting that movement of the voltage sensors is much faster than the allosteric closed-open isomerizations. A related model was also used to perform energetic considerations of HCN2 channels [39]. MWC models were also applied previously to other members of the superfamily of tetrameric voltage-gated channels, including Shaker channels [40] or *m*Slo channels [41] and Ca^++^ channels [42].

Progress in the understanding of voltage-dependent gating of HCN channels has been achieved by combining two types of measurement in related spHCN channels in parallel, channel activation by voltage-clamp and conformational changes of the S4 segment by fluorescence intensity [43]. When fitting their data with a related 10-state MWC model, these authors obtained a different result: The S4 motion is not fast but rate limiting for channel opening. Moreover, the results suggested that spHCN channels open after only two S4 segments have moved and that the action of the voltage sensors is not cooperative. However, spHCN channels are functionally not entirely representative for HCN channels because a pronounced slow mode shift [44] is only minor in mammalian channels [45].

In contrast to this, our previous results upon the cAMP-induced activation in homotetrameric HCN2 channels showed a pronounced and surprising type of cooperativity for the subunit action. Studying cAMP binding and activation gating in parallel by confocal patch-clamp fluorometry [46, 47], we determined by global fit strategies a full set of rate and equilibrium constants in a Markovian model related to the 10-state MWC model. This allowed us not only to consider the action of the subunits in time but also to gain insight into the sequence of affinities of the four binding steps. The result was a surprising sequence of cooperativity of positive, negative and positive for the second, third and fourth binding step, respectively [48].

At the first glance the antipode between a non-cooperative voltage-dependent gating, with the involvement of only two subunits, and the highly cooperative ligand-induced gating, with the sequence positive—negative—positive, is not implausible if taking into account that different parts of the channel govern these two processes, the transmembrane core portion presumably evokes the voltage-induced gating whereas the cytosolic CNBD evokes that ligand-induced gating. However, we previously proved for HCN2 channels a significant reciprocal relationship between voltage- and ligand-induced gating by demonstrating that not only cAMP binding enhances channel activation but also voltage-induced activation enhances cAMP binding, indicating an intimate coupling between both processes [47]. To shed more light into this dual gating, it is required to determine also for the voltage-induced gating a kinetic scheme in similar detail as determined previously for the ligand-induced gating [48–50].

In this study, we globally analyze a set of multiple time courses of activation and deactivation of HCN2 channels over a wide voltage range in both the absence and presence of cAMP and we use Markovian state models to unravel the molecular operation of the channel. We show that the activation gating of HCN2 channels is evoked by two separable voltage steps followed by a voltage-independent pore opening. Furthermore, we identify specific effects of cAMP on these individual steps and we show that cAMP reduces the effective gating charge. Based on our models we calculate gating currents at different voltages and we discuss the results in the context of a structure reported for related HCN1 channels recently [31].

## Results

### Time courses of the open probability

**Fig 1:**
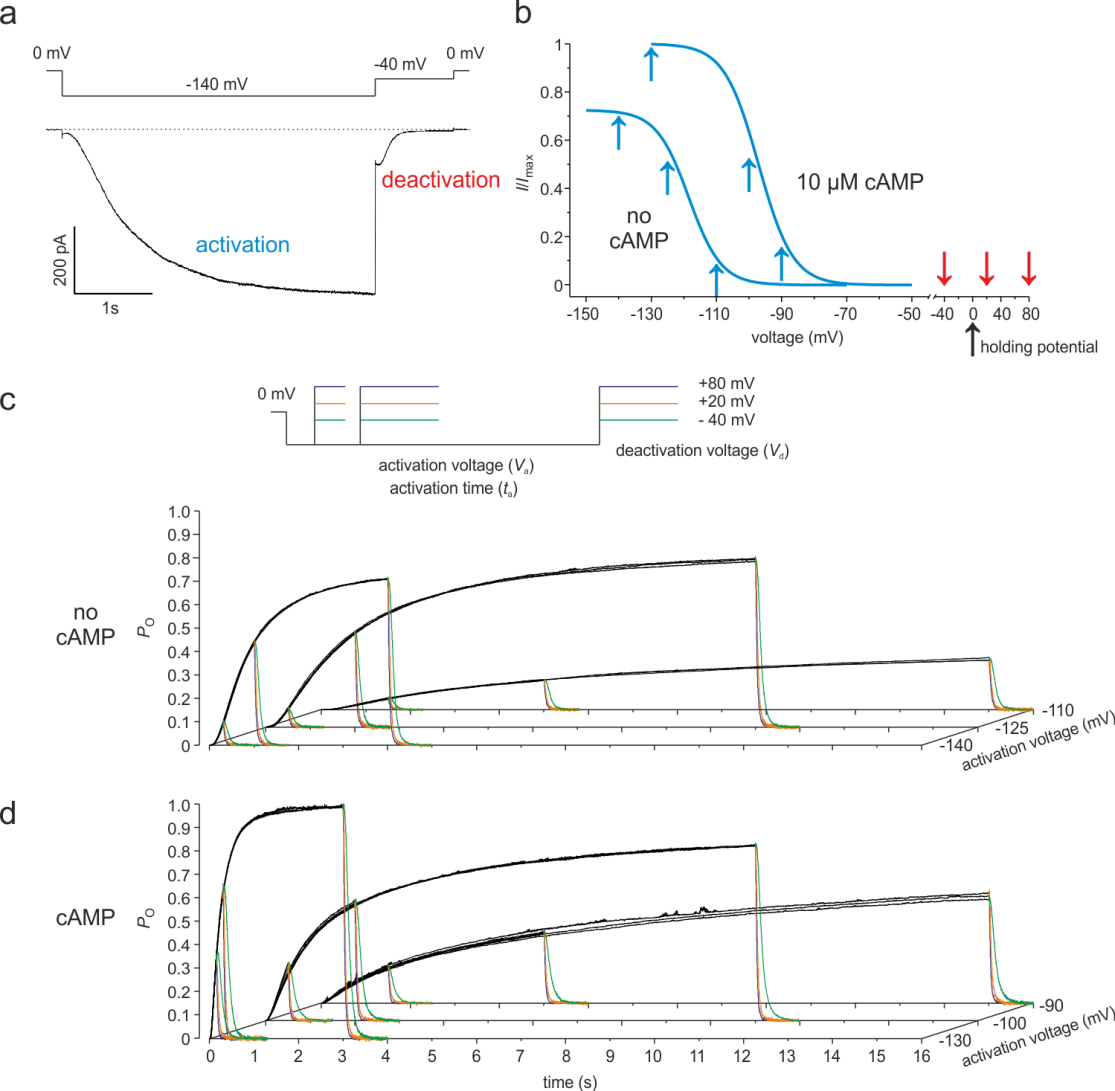
Multiple time courses of *P*_o_ covering a wide voltage range for HCN2 channel gating. Currents were activated by hyperpolarizing pulses to the voltage *V*_a_ with the duration *t*_a_ followed by depolarizing pulses to the voltage *V*_d_ to generate deactivation. (**a**) Representative current trace. *V*_a_ = - 140 mV, *t*_a_ = 4 s, *V*_d_ = -40 mV. (**b**) Steady-state activation in the absence of cAMP and in the presence of 10 μM cAMP. The Boltzmann curves, obtained with pulses of 4 s duration, were computed according to a previous report by equation S1 [47]. The values were: no cAMP: *z* = 5.15, *V*_h_ = -118.4 mV; 10 μM cAMP: *z* = 4.88, *V*_h_ = -97.1 mV. The blue and red arrows indicate the used values of *V*_a_ and *V*_d_, respectively. (**c**) Set of *P*_o_ traces following 27 different double pulse protocols in the absence of cAMP. *V*_a_ = -140 mV: *t*_a_ = 0.3 s, 1 s, 4 s; *V*_a_ = -125 mV: *t*_a_ = 0.5 s, 2 s, 11 s; *V*_a_ = -110 mV: *t*_a_ = 1.5 s, 5 s, 15 s (n = 5–18). (**d**) As c but in the presence of cAMP. *V*_a_ = -130 mV: *t*_a_ = 0.15 s, 0.3 s, 3 s; *V*_a_ = -100 mV: *t*_a_ = 0.5 s, 2 s, 11 s; *V*_a_ = -90 mV: *t*_a_ = 1.5 s, 5 s, 15 s. In c and d *V*_d_ was -40 mV, 20 mV, 80 mV (n = 7–10).

HCN2 channels were expressed in *Xenopus laevis* oocytes and the time courses of voltage-dependent activation and deactivation (Fig 1A) were studied in both the absence of cAMP and the presence of 10 μM cAMP, a concentration generating a saturating effect in both activated and non-activated channels [51]. In an attempt to systematically sample a wide effective voltage range for both channel activation and deactivation, we applied 27 double pulse combinations, differing by activation voltage, activation time and deactivation voltage. According to previous results [47], the activation voltages in the absence of cAMP were set to -140, -125 and -110 mV whereas in the presence of cAMP the respective voltages were -130, -100 and -90 mV (blue arrows in Fig 1B). Deactivation was measured under both conditions at -40, +20 and +80 mV (red arrows in Fig 1B).

In order to fit Markov state models to the macroscopic time courses of activation and deactivation, the macroscopic currents were transformed into time courses of open-state probability, *P*_o_ [52] (see Supplementary Methods S1 Text). To gain smoother time courses of *P*_o_, currents from 5 to 18 patches were recorded for each of the 27 pulse combinations and the respective time courses were averaged. This approach provided us 27 reasonably smooth time courses of *P*_o_ for both the absence and presence of cAMP (Fig 1C and 1D). These time courses were subjected to the following global fit analysis. Notably, in none of the models tested herein transitions for a slow mode shift [44] were included because in HCN2 channels these mode shifts are only small [45].

### Activation gating in the absence of cAMP is caused by two distinguishable steps

**Fig 2:**
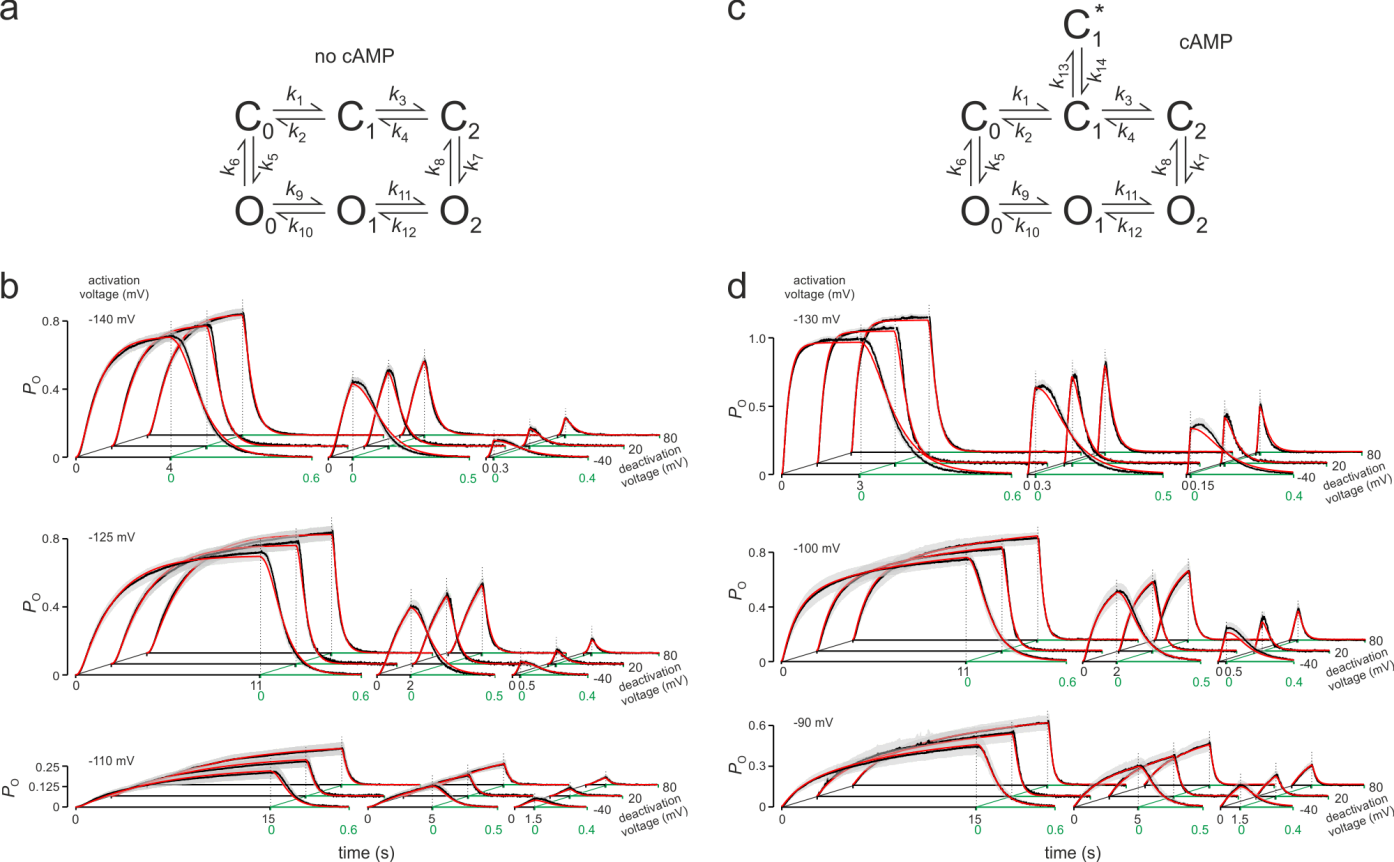
Global fit of activation and deactivation time courses in either the absence or presence of cAMP. (a) Scheme of model 1_n_. (b) Fit of the 27 time courses of activation and deactivation in the absence of cAMP. (c) Scheme of model 1_a_. (d) Fit of the 27 time courses of activation and deactivation in the presence of 10 μM cAMP. The experimental traces and fitted curves are given in black and red color, respectively. Shades of gray indicate s.e.m. The parameters are provided by Table 1.

To demonstrate the adequacy of the global fit with Markov models, the 27 time courses of *P*_o_ in Fig 1C were rearranged, improving visual discrimination of each individual averaged time course (Fig 2, black traces). We first fitted the allosteric model 14_n_ proposed by Altomare and coworkers [38] containing fixed stoichiometric factors for the four gating steps (st), four voltage-dependent steps with equal gating charge z (ze), and a constant allosteric factor (f) (S1A Fig; S1 Table). Inspection of the fit by eye revealed that the model basically describes the measured time courses but had also notable deficiencies. The range of s.e.m. for each averaged trace is indicated by the shade of gray (S1B Fig). The residual sum of squares (*RSS*) and the normalized mean standard error (MSE^*^; S1 Text), used for our model ranking, were large. Five modified models of the Altomare type with between 5 and 9 parameters produced similar deficiencies.

We then systematically tested a large number of other models and used MSE^*^ as ranking parameter. In all tested models the closed-open isomerizations were assumed to be a one-step process that is voltage independent [53] and it turned out that, as in the Altomare model, deactivation required consistently a pathway differing from that in activation, running first along the open states until the channel closes. We also tested the effects of relieving the constraints set by a fixed stoichiometric factor (st), equal gating charges in all steps of either the closed or open channel (ze) and a fixed allosteric factor (f). In all approaches we tested if the models can reach a smaller MSE^*^ value than that reached by model 14_n_ proposed by Altomare and coworkers. Smaller MSE^*^ values were reached by 13 models summarized in Supplementary Table (S1 Table). Because voltage-dependent gating of HCN channels has been described repeatedly as a two-step [18, 43, 54] and even a one-step process [53] we also included Markov models with less than four voltage-dependent steps. Indeed, models with either three, two and even one voltage-dependent step were superior over the Altomare model. Furthermore, we also included in our analysis coupled dimer (CD) models which assume that a channel is open when both dimers are in an open state [54]. The overall result was that model 1_n_ with two gating steps and none of the constraints st, ze and f (Fig 2A and 2B) superior over all others followed by the respective CD model 2_n_ (S1 Table). Notably, these models produced a similar amount of summed gating charge z (sumz) of about 8 and 7, respectively. The fact that in model 1_n_ two parameters were not determined, can be ignored because they belong to extremely small rates for the activation along C_0_→O_0_→O_1_→O_2_, indicating that this activation pathway is not employed. The models 3_n_ to 14_n_ were not further considered because the number of non-determined parameters was either too high or the fit quality was too poor or both. Together our results suggest that two subunits play a key role in channel activation, matching a previous result in related spHCN channels [43].

### Also at saturating cAMP activation is governed by two distinguishable voltage-dependent steps

The 27 current traces recorded by applying double pulses in the presence of cAMP (Fig 1D) were subjected to the same type of analysis as the 27 traces without cAMP (S3 Table). A simple approach was to keep model 1_n_, as well as all rate constants obtained without cAMP, and to introduce the cAMP effect by modifying some of them in a reasonable way. Based on single-channel recordings *P*_o_ was set to 0.99 [52]. Three fits were performed: 1.) Only the opening rates were accelerated by a factor *A*. 2.) Like 1. but the rates for voltage activation were additionally accelerated by the factor *B*. 3.) The rates for voltage activation were accelerated by a factor *B*, the rates for voltage deactivation were decelerated by *B*^-1^. Reasonable fits were not obtained. Then the constraints in the global fit with model 1_n_ were considerably relieved: All rate constants of the closed-open isomerizations and all voltage-dependent rate constants at zero voltage were treated as free parameters, keeping only *z*_1_ and *z*_2_ from the fit without cAMP. Including further *P*_o_ = 0.99 and detailed balance in the 1_n_ model, this results in 10 free parameters. The result was that even with this large number of free parameters a reasonable fit was not obtained (S2 Fig).

We therefore assumed that the gating is profoundly changed by cAMP and we fitted a variety of models with one to four voltage-dependent steps without and with closed-open isomerizations at partial activation. Similar to the absence of cAMP, five models with either two, three or four gating steps (models 5_a_-7_a_, 10_a_, 11_a_) were superior over the Altomare model (model 13_a_). Despite this superiority these models showed a consistent deficit in describing the slow activation at moderate hyperpolarization (c.f. S1C Fig). To overcome this we tested models with an additional step from an intermediate closed state to another closed state either within or outside the activation pathway. While an additional step within the activation pathway was clearly inadequate, an additional transition C_1_↔C_1_^*^ outside the activation pathway did improve the fit by describing the slow activation time courses at moderate hyperpolarization. When further assuming that C_1_↔C_1_^*^ depends on voltage, the quality of the fit was further improved, resulting in the best models 1_a_-3_a_ (S3 Table). As in the absence of cAMP, and as expectable, the best fits were obtained by relieving the constraints given by a fixed stoichiometric factor (st), equal gating charges in all steps of either the closed or open channel (ze) and a fixed allosteric factor (f). It is notable that the summed gating charge for all three models 1_a_-3_a_ was closely similar with a value some above 5 although model 3_a_ contained even three instead of two gating steps. This suggests that the number of gating charges does not hypercritically depend on the specific model. The models 4_a_ to 13_a_ were not further considered because the number of non-determined parameters was either too high or the fit quality was too poor or both. Model 1_a_ was outstanding because the fit was best (Fig 2C and 2D) and all parameters were determined.

### Attributing the effects of cAMP to specific model parameters

As outlined above the first effect of cAMP on the voltage-dependent gating is to generate accessibility to an additional closed state C_1_^*^ in the activation pathway which is not accessible in the absence of cAMP. Beyond this effect, the sameness of the remaining structure of model 1_a_ with model 1_n_ allowed us to identify specific effects of cAMP on the individual parameters of these models. A second effect of saturating cAMP is an about 40-fold increase of *E*_2_ = *k*_7_/*k*_8_, resulting in a marked shift of the equilibrium C_2_-O_2_ to O_2_.

The third effect of cAMP on the voltage-dependent rate constants is more complex, affecting both the gating charges *z*_x_ and the rate constants at zero voltage, *k*_x_^0^, in a specific way (Table 1): While *z*_1_ is kept fairly constant (0.78 versus 0.79) *z*_2_ is approximately halved (4.34 versus 7.31), resulting in a decrease of the total gating charge sumz = *z*_1_+*z*_2_ from 0.79+7.31 = 8.10 in the absence of cAMP to 0.78+4.34 = 5.12 in the presence of cAMP. The combination of facts that the data with cAMP could not be reasonably fitted with the gating charges obtained in the absence of cAMP (c.f. Fig 2) and that *z*_1_ is unchanged in the fits with the 1_n_ and 1_a_ model without cAMP (1_n_ model) and with cAMP (1_a_ model) strengthens the conclusion that cAMP has a specific effect on the second but not on the first voltage-dependent gating step. To further consolidate the effect of cAMP on *z*_2_, we also fitted model 1_a_, with *z*_1_ and *z*_2_ fixed to the values determined by the 1_n_ model, resulting in a fit with 13 free parameters. Though this fit was improved with respect to using the 1_n_ model, it did not reach the quality obtained by using model 1_a_ with *z*_1_ and *z*_2_ being free parameters and, most importantly, all three rate constants leaving C_1_ were undetermined. Therefore, this fit was not further considered.

For the *k*_x_^0^ values, the effects of cAMP result in characteristic voltage dependencies of the 8 effective rate constants of the four voltage-dependent steps (S3 Fig). Concerning the ON-rate constants, the cAMP effects are heterogeneous: In the closed channel there is a shift to higher rates for *k*_1_ at all voltages whereas for *k*_3_ the stronger voltage dependency becomes flattened, resulting in a higher cAMP effect at depolarized potentials. Concerning the OFF-rate constants, there is nearly no cAMP effect on the first gating step, neither in the closed (*k*_2_) nor open channel (*k*_10_). In contrast, cAMP flattens the voltage dependence of *k*_4_ and *k*_12_, specifying the second gating step in the closed and open channel, respectively, thereby crossing the relationship in the absence of cAMP at -50 mV. Together, these results show that there is not only an enhanced voltage dependence of the second compared to the first gating step in the absence of cAMP, but also that cAMP predominantly decreases the voltage dependence of the second gating step by reducing *z*_2_.

**Table 1:** Parameters for the fits with models 1_n_ (no cAMP) and 1_a_ (10 μM cAMP).

		model 1_n_ (no cAMP)		model 1_a_ (10 μM cAMP)	
No.	parameter	value	se in %	Value	se in %
1	*k*_1_^0^	1.60×10^−1^	7	3.24	8
2	*k*_2_^0^	5.01×10^1^	13	1.21×10^2^	38
3	*z*_1_	0.79	3	0.78	4
4	*k*_3_^0^	8.12×10^−8^	22	1.70×10^−3^	39
5	*k*_4_^0^	3.32×10^6^	13	9.88×10^4^	23
6	*z*_2_	7.31	1	4.34	3
7	*k*_5_	1.77×10^−2^	56	2.12×10^−1^	11
8	*k*_7_	3.11	3	4.45	1
9	*k*_9_^0^	1.30×10^−3^	n.d.	3.10	30
10	*k*_10_^0^	2.35×10^1^	2	2.68×10^1^	2
11	*k*_11_^0^	5.11×10^−5^	n.d.	4.92×10^−2^	59
12	*k*_12_^0^	8.93×10^3^	6	6.96×10^2^	10
13	*k*_13_^0^			1.85×10^−1^	37
14	*k*_14_^0^			1.84	26
15	*z*_c_			1.03	12

All rate constants, *k*_x_, are given in s^-1^. The effective gating charges, *z*_x,_ are dimensionless. The standard errors of the parameters, *se*, were obtained by equation S5. *k*_6_ and *k*_8_ were obtained from microscopic reversibility and *k*_7_×(1-*P*_o,sat_)/*P*_o,sat_ to be 2.93×10^1^ s^-1^ and 1.26 s^-1^ in the absence of cAMP and 3.80×10^1^ s^-1^ and 4.49×10^−2^ s^-1^ in the presence of cAMP, respectively (S2 Table). n.d. means ‘not determined’ (s.e. >60%).

### Time-dependent population of states

**Fig 3:**
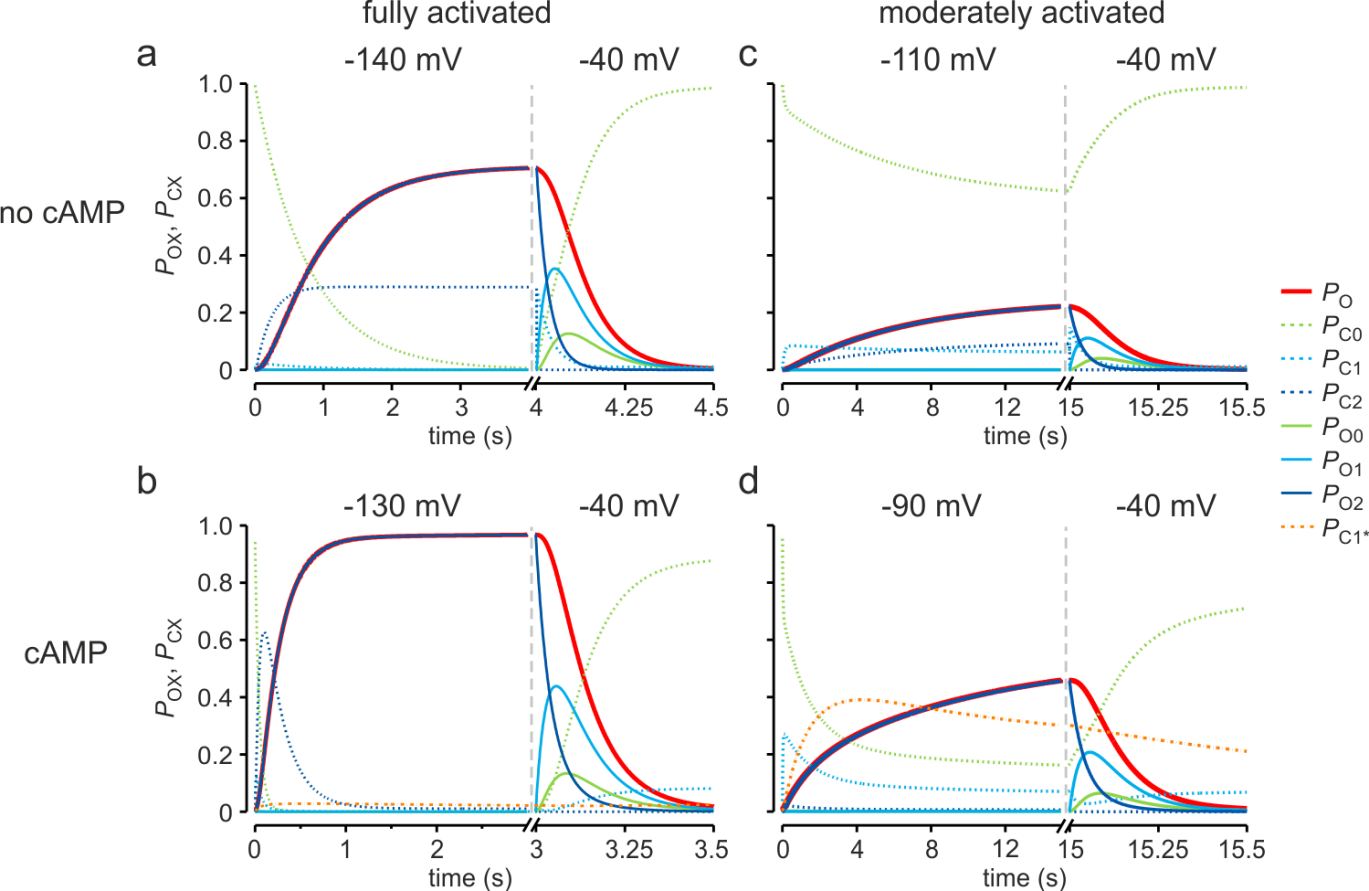
Effect of cAMP on the time-dependent population of states. The time courses were computed with model 1_n_ (no cAMP) or 1_a_ (10 μM cAMP). (**a**,**b**) Full activation and deactivation without and with cAMP. (**c**,**d**) Moderate voltage-dependent activation and deactivation without and with cAMP.

We next considered the time-dependent population of all states for the selected models 1_n_ and 1_a_ (S1 Text). At full voltage-dependent activation and no cAMP, relevantly occupied states are C_0_, C_2_, and O_2_: C_0_ is slowly emptied and the equilibrium C_2_-O_2_ becomes populated accordingly, generating a *P*_o_ of 0.71 (Fig 3A). At full voltage-dependent activation in the presence of cAMP, C_0_ is emptied rapidly to O_2_ along the closed states with a *P*_o_ of 0.99 (Fig 3B). The buffer state C_1_^*^ is practically not populated. At moderate voltage-dependent activation and no cAMP, C_0_ is emptied very slowly and dominates the filling of O_2_, but also of C_2_ and C_1_ (Fig 3C). At moderate voltage-dependent activation and in the presence of cAMP the situation differs: The slow depopulation of C_0_ is followed by a transient population of C_1_ and, subsequently, by an articulate filling of the buffer state C_1_^*^ (Fig 3D). The slow emptying of C_1_^*^ causes the slow activation phase typical for moderate voltage-dependent depolarization in the presence of cAMP. Deactivation from O_2_ runs preferentially through the other open states, generating the tail currents with their characteristic delay.

### Time-dependent probability flux densities

**Fig 4:**
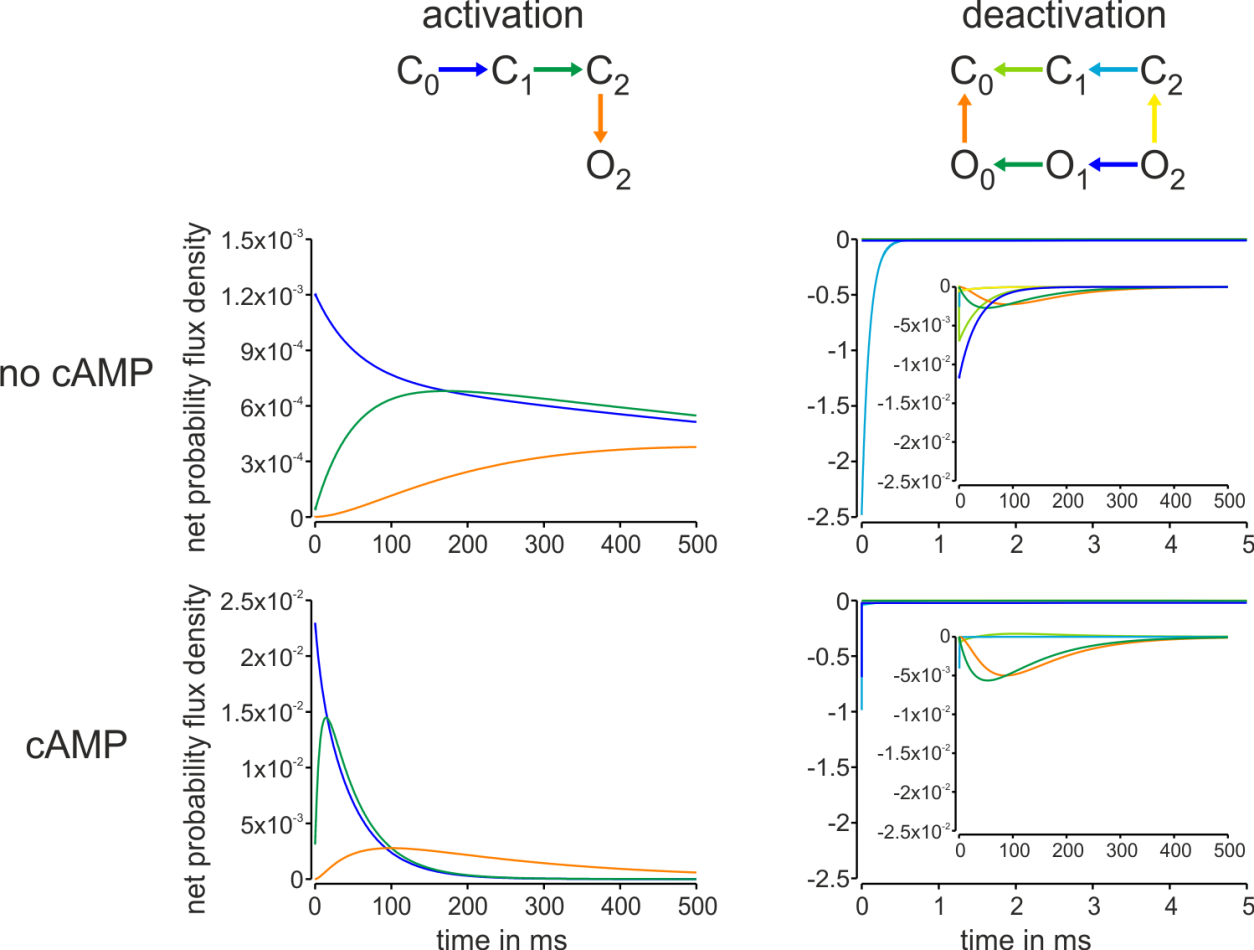
Effect of cAMP on the probability flux densities along the main pathways of activation from 0 mV to -130 mV and subsequent deactivation to -40 mV. The time courses were computed with model 1_n_ (no cAMP) or 1_a_ (10 μM cAMP). The colors of the arrows in the top schemes correspond to the colors of the probability flux density time courses. The predominant effect of cAMP is to strongly accelerate the probability flux density of the activation pathway.

From the time-dependent population of states we computed the cAMP effects on the time-dependent probability flux densities for the individual steps of the models 1_n_ and 1_a_. In the absence of cAMP, the probability flux density along the relevant activation pathway C_0_→C_1_→C_2_→O_2_ is slow and still incomplete after 500 ms (Fig 4, left). Notably, cAMP enlarges the peak probability flux density about 20 fold and accelerates its time course respectively. This suggests that cAMP binding facilitates the action of the voltage sensor domain. Upon deactivation, in the absence of cAMP the probability flux densities show two components, a very fast component from C_2_ to C_1_ and slow components from O_2_ to C_0_ through the other open states and from C_1_ to C_0_. In the presence of cAMP the slow component from O_2_ to C_0_, approximately mirroring the kinetics of pore closure, dominates the time course nearly alone (Fig 4, right). This is because at -130 mV the probability to be in O_2_ is close to unity. The kinetics of the slow component is essentially not affected by cAMP.

### Computed gating currents

**Fig 5:**
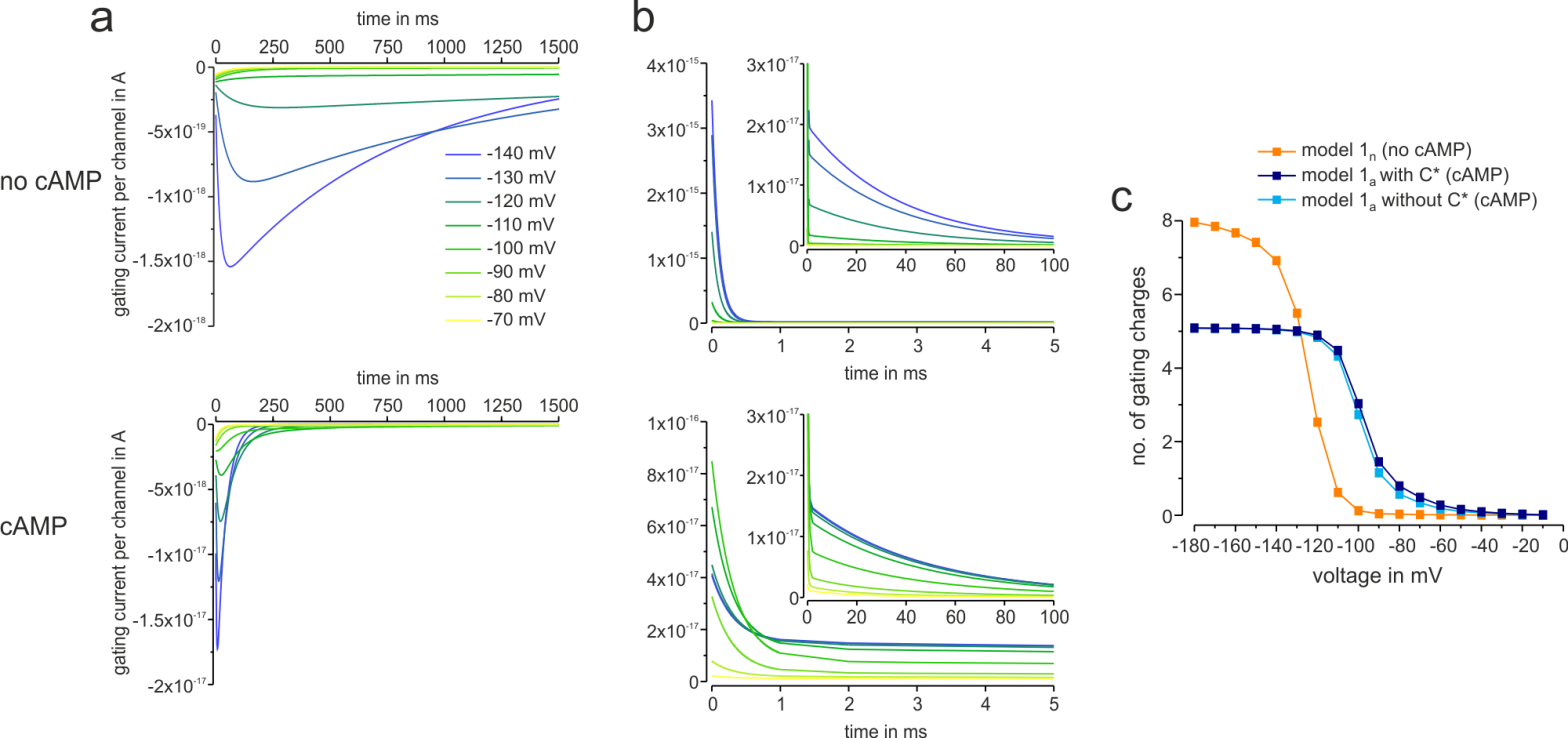
Effect of cAMP on computed gating currents per channel and moved gating charges. The gating currents per channel were computed with model 1_n_ (no cAMP) or 1_a_ (10 μM cAMP). The holding potential was set to 0 mV. (**a**) ON-gating currents at the voltages between -140 and -70 mV. The main effect of cAMP is to significantly accelerate both the rising and the decay phase of the gating current, resulting in an increase of the peak amplitude. (**b**) OFF-gating currents at -40 mV following voltage pulses indicated in (a) containing a fast and a slow component. Both components are decreased by cAMP. (**c**) Effect of cAMP on the total gating charge computed by integrating the gating currents for a voltage pulse of 1.5 s duration in the absence and presence of cAMP. The holding potential was set to 0 mV. The effect of the voltage-dependent transition C_1_→C_1_^*^ is only minor.

For a single voltage-dependent step the product of the time-dependent probability flux density, the gating charge *z*_x_ and the elementary charge, *e* = 1.602×10^−19^ C, determine the gating current provided by this step. Forming these products for the four voltage-dependent steps C_0_ to C_2_ and O_0_ to O_2_ in either model 1_n_ or 1_a_ and summing up these products for each model results in the time course of the total gating current provided by these steps. It should be noted that the gating currents computed in this way describe only the moved charges finally acting on the pore opening but not necessarily all moved charges. The contribution of the C_1_-C_1_^*^ transition can be ignored in the following consideration because *z*_c_ is only 1.03 (Table 1) and the time course is extremely slow. Corresponding to the probability flux densities, the computed gating currents upon activation are slow in the absence of cAMP, reaching at -140 mV a peak value of ~1.5×10^−18^ A per channel, whereas cAMP accelerates the gating current and enlarges its amplitude at the same voltage by ~11 fold to a peak value of ~1.7×10^−17^ A per channel (Fig 5A).

Upon deactivation, the computed gating current is biphasic: The fast component is reduced by cAMP about 40 fold, due to the much lower occupancy of C_2_. The slow component, mirroring the time course of pore closure, has an approximately unchanged kinetics. Its amplitude is some smaller because of the smaller summed gating charge *z*_1_+*z*_2_ although O_2_ is more populated (Fig 5B).

Plot of the moved gating charges for a pulse of full voltage-dependent activation illustrates that cAMP reduces the number of gating charges from 8.10 to 5.12 (Table 1) but also shifts the voltage dependence of this movement to more depolarized potentials (Fig 5C), thereby enabling activation at less hyperpolarizing potentials compared to the absence of cAMP.

## Discussion

In this study we provide insight into the voltage-induced gating kinetics of HCN2 channels in both the absence and presence of cAMP. Our analyses are based on the models 1_n_ and 1_a_ for the condition ‘no cAMP’ and ‘saturating cAMP’, respectively (c.f. Fig 2). These models differ by one structural aspect: Model 1_a_ contains an additional transition C_1_-C_1_^*^ to include a slow activation component at moderate hyperpolarization in the presence of cAMP. This difference, however, does not hinder direct comparison of the parameters in models 1_n_ and 1_a_ because it can be readily assumed that model 1_n_ also includes the C_1_-C_1_^*^ transition but is not recruited. Our results show: (1) Voltage-evoked activation of HCN2 channels is governed by two kinetically distinguishable voltage-dependent steps. (2) In voltage-dependent activation there is pronounced cooperativity between the subunits in both the absence and presence of cAMP: The second gating step is associated with a shift of nine to five times more gating charge than the first gating step, respectively. (3) cAMP characteristically modulates the cooperativity of the two gating steps by specifically reducing the gating charge *z*_2_ of the second gating step. (4) cAMP also affects the voltage-independent closed-open isomerization C_2_-O_2_ by shifting this equilibrium to O_2_.

Together, this study provides quantitative information about the activation of HCN2 channels in both the absence and presence of cAMP.

### Activation of HCN2 channels is governed by a two-step process

The result that two voltage-dependent steps reasonably describe activation basically matches previous results in related spHCN channels [43]. The close structural similarity between our models 1_n_ and 1_a_ allowed us to directly compare the cAMP effects on the parameters of the two-step models which is considered below in the context of structural results. Beyond this, our analysis enables us to compute true, otherwise not accessible, steady-state relationships and, moreover, to consider to what extent insufficiently long pulses distort measurements of the steady-state concentration-activation relationships. In the absence of cAMP usage of model 1_n_ yields that pulses of 1 and 4 seconds duration were much too short to approximate the steady-state relationship whereas pulses of 15 seconds duration approximated the steady-state relationship reasonably (S4A Fig). In the presence of cAMP the channels gate faster and, again, voltage pulses of 15 seconds duration did not fully match the computed steady-state relationship (S4B Fig). Hence, true steady-state concentration-activation relationships would certainly require sequences of voltage pulses with durations of a minute or even longer and sufficiently long recovery intervals between the pulses. However, in inside-out patches, the only condition allowing for a control of the cytosolic cAMP concentration, this is practically impossible due to run-down phenomena typical for HCN channels [55]. Hence, model-based approaches as presented herein can provide a guess to understand channel activation when equilibrium cannot be reached in the experiments.

### Gating charge and gating currents

The total gating charge *z*_1_+*z*_2_ in the presence of cAMP was 5.12 which is clearly smaller than 8.10 in the absence of cAMP (Fig 5C). At the first glance this significant reduction is surprising because the steady-state activation relationship with cAMP is apparently similarly steep to that without cAMP (c.f. Fig 1B). One should be aware, however, that in schemes of coupled steps the equilibrium of an individual step is not independent of the neighbored steps, as insightfully demonstrated previously [56]. Hence, the effect of cAMP to shift the equilibrium C_2_-O_2_ strongly to O_2_, must influence also the adjacent voltage-dependent gating steps.

The gating currents computed by our models 1_n_ and 1_a_ (c.f. Fig 5A and 5B) showed specific effects of cAMP: Upon activation, the ON-gating currents in the presence of saturating cAMP are about 11 times larger in their peak amplitude and respectively faster. Hence, cAMP has a strong accelerating effect on the speed of movement of the gating charges. The fact that the total gating charge is reduced by cAMP from ~8 to ~5 is thereby subordinate. Upon deactivation, cAMP had no major effect on the time courses of the fast and slow current component; only the amplitude of the fast component was markedly reduced which can be interpreted in the most plausible way by a stronger population of the state O_2_ and a corresponding lower population of C_2_.

When considering the question whether the gating currents as calculated herein can be measured experimentally, one has to state that they are exceptionally small in amplitude because of their general slowness, in particular the ON-gating currents in the absence of cAMP. If one assumes the best available technique to record gating currents, the cut-open oocyte technique, an optimistic estimate of 10^8^ channels per oocyte and a resolution of 50 nA, one could only expect to get a correlate of the rapid OFF-gating current component in the absence of cAMP but not of the subsequent slower component. However, in cut open-oocytes the actual cAMP concentration cannot be fully controlled. Accordingly, this would inevitably lead to another serious technical problem: If the cAMP concentration only minimally changes during the measurement, subtraction of linear capacitive current components would become impossible. It should be noted that in related spHCN channels gating currents were indeed measured with the cut-open oocyte technique [44]. The activation gating of spHCN channels is generally faster than that in HCN2 channels. Accordingly, the gating currents in spHCN channels were complete for both the ON- and the OFF-gating after approximately 80 ms. A slow component and a dependence on cAMP has not been reported. Together, our indirect approach seems to be a helpful approach to gain insight into the gating currents produced by the slow HCN2 channels.

### Mechanistic interpretations based on structural data

The recently published structure for a full HCN1 channel by cryo-electron microscopy provides detailed information about the steric relationship between the voltage-sensor domain, CNBD and the pore region [31]. Concerning the combined activation gating by hyperpolarizing voltage and cAMP the authors proposed the following scenario: At depolarization, the unusually long S4-helices extend into the cytosol and contact there part of the C-linker, stabilizing the closed gate formed by the inner part of the four S6-helices. The closed gate becomes further stabilized by a packing arrangement of S4, S5 and S6 helices of each subunit and by an HCN domain. Hyperpolarization induced activation is thought to be evoked by a downward displacement of the S4 helices, disrupting these stabilizing interactions, allowing the S6 helices to spontaneously open. The activating effect of cAMP has been attributed to local conformational changes propagating to the channel and initiating a rotation, thereby promoting opening of the inner gate.

**Fig 6:**
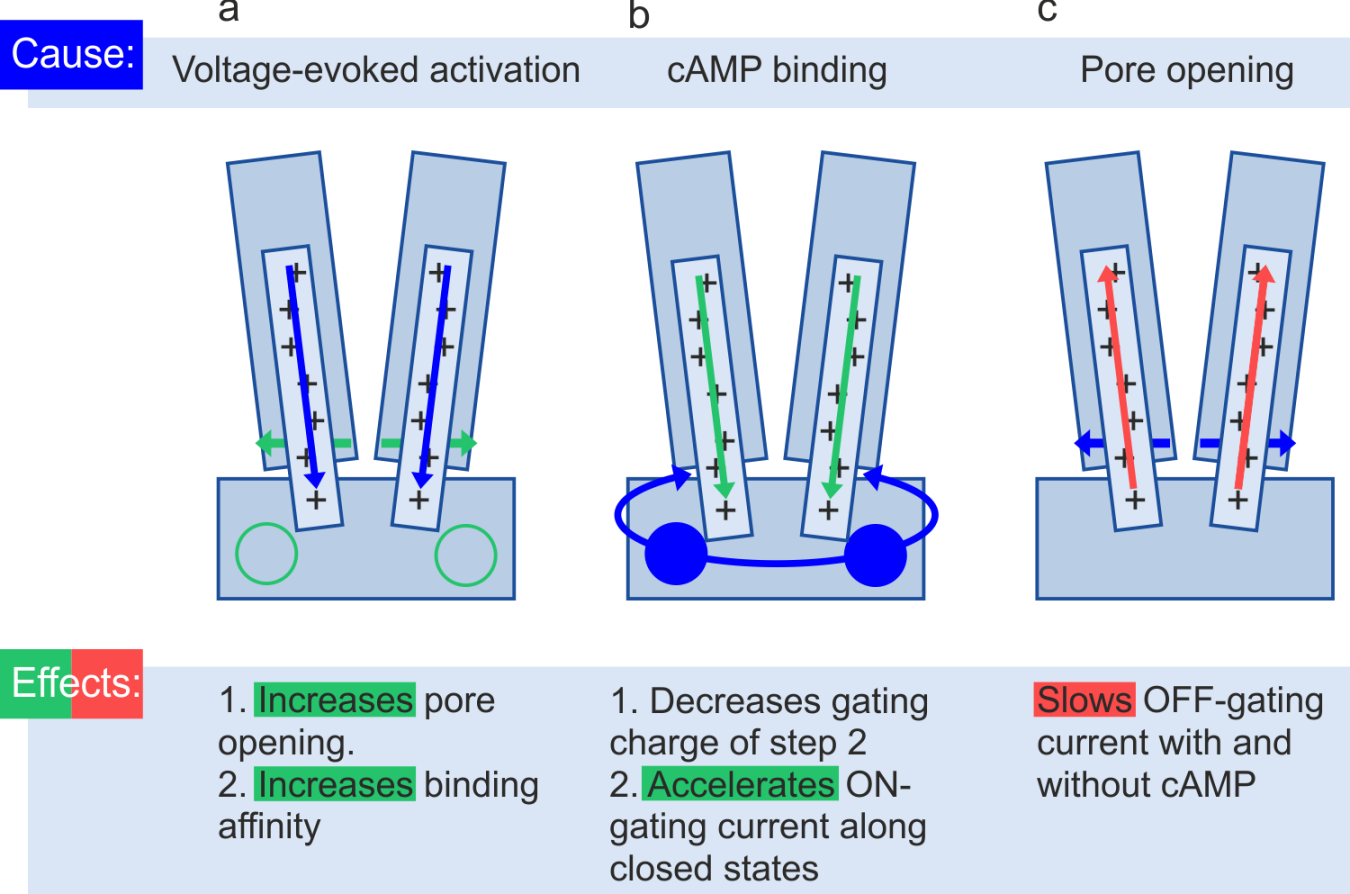
Cartoons illustrating the mutual interactions between mutual stimuli. The graphs show schematically two S6-helices forming the inner gate at their inner end, two S4-helices being the voltage sensor and the CNBD with two of the four binding sites (circles). Blue color symbolizes a cause, green and red color an activating and inhibiting effect, respectively. **(a)** Effects of voltage-evoked activation on binding affinity and *P*_o_. **(b)** Effects of cAMP binding on gating charge and ON-gating current. **(c)** Effects of pore opening on OFF-gating current. For further explanation see text.

How can our new functional results in the context with previously published functional results be related to this scenario? Because HCN channels are activated by hyperpolarizing voltage it is *a priori* clear that the moved VSDs, including the S4-helix, open the gate at the inner end of the S6-helices (Fig 6A). By combined measurements of ligand binding and activation gating with confocal patch-clamp fluorometry we previously identified a second effect of voltage activation, namely to enhance the binding affinity for cAMP by about three fold [47]. And, based on the result that at hyperpolarizing pulses this enhancement of the binding affinity precedes current activation, we demonstrated that this enhancement of the binding affinity is caused by a direct interaction of the VSD with the CNBD, bypassing the pore (Fig 6A).

If there is a direct interaction between the CNBD and the VSD it must also work in the opposite direction. Therefore, the reduction of the gating charge by cAMP observed in the present study suggests that the rotation of the CNBD pulls the VSD, with part of the charges of the S4-segments, out of the membrane into the cytosol, thereby reducing the amount of charges remaining in the electric field across the membrane and, at the same time, promoting activation (Fig 6B). The pronounced acceleration and enlargement of the ON-gating current by cAMP can be interpreted by facilitating the inward movement of the S4-helix (Fig 6B). Another result of the present study can be explained easily in this context: The open channel has a much slower OFF-gating current than the closed channel (c.f. Fig 5B), resulting from the much faster transition C_2_→C_1_ than the transitions O_2_→O_1_→O_0_ (c.f. Fig 4B). It is therefore likely that the open pore domain sterically hinders movement of the S4-helix in the outward direction in both the absence and presence of cAMP (Fig 6C).

To discuss the mechanism why cAMP does not change the small effective gating charge *z*_1_ of the first step but notably reduces the gating charge *z*_2_ of the second step, the most plausible and simple interpretation seems to be the following: 1.) The first gating step with *z*_1_ = ~0.8 proceeds independent of the position of the gating ring at either zero or saturating cAMP. 2.) In the absence of cAMP ~7.3 gating charges are effectively moved for full voltage-dependent activation. 3.) The first gating step is not only a prerequisite of the second gating step but also for the effect of cAMP. 4.) In the presence of saturating cAMP, the gating ring is turned such, that the number of charges in the electrical field is reduced from ~7.3 to ~4.3 by either pulling the S4 helix into the cytosol or by forming watery crevices around the VSD. 5.) The acceleration of activation at reduced effective gating charge in the presence of cAMP can be explained by a reduced intensity of the molecular interaction between the S4 helix and its environment (*k*_3_ is accelerated over the whole voltage range see S3 Fig).

To further unravel the complex temporal relationships between the two activating stimuli, it seems to be an attractive idea for future experiments to quantify extent and kinetics of the S4-movement upon channel activation by an appropriate fluorometric approach, ideally in parallel with optical recording of ligand binding.

## Materials and methods

### Ethics statement

The obtainments of the oocytes were done under permission of the Thüringer Landesamt für Verbraucherschutz (Reg.-Nr.: 02-037/13).

### Preparation of oocytes and RNA injection

The oocytes were obtained from adult *Xenopus leavis* under anesthesia (0.3% 3-aminobenzoic acid ethyl ester) and injected with cRNA encoding wild type mHCN2 channels of *Mus musculus* (NM_008226) as described previously [52].

### Electrophysiology

Currents were recorded with standard patch-clamp techniques in inside-out macropatches obtained from the oocytes expressing the channels (S1 Text).

### Data analysis and computations

The global fit strategies, time-dependent population of model states, net probability flux densities and gating currents are described in S1 Text.

Experimental data are given as mean ± s.e.m. The treatment of standard errors obtained by the fits is described by equation S5 in S1 Text.

## Supporting information

S1 Text Supplementary methods. (DOCX)

S1 Table Models used to fit the data in the absence of cAMP. C_x_ and O_x_ mean closed and open states respectively. In coupled-dimer models the allosteric step leads to the flipped state F_x_. Opening appears only if both dimers are in a flipped state. This is indicated by the index CD→O. st, stoichiometric factors used; ze, gating charge z equal in all steps, f, allosteric factor used; sumz, sum of gating charges; p, number of parameters; nd p, number of parameters not determined given by a standard error >60%, *RSS*, residual sum of squares of all 27 traces; MSE^*^, normalized mean square error given by equation S7; rX_a_, indicates corresponding models in S3 Table. CD means coupled dimer.
(DOCX)

S2 Table Calculation of rate constants for models 1_n_ (no cAMP) and 1_a_ (10 μM cAMP). From the fit parameters (Table 1) the rates (s^-1^) were determined as listed. *P*_o,sat_ was set to 0.71 and 0.99 in the absence and presence of cAMP, respectively [47, 52]. *indicates rate constants used only in model 1_a_.
(DOCX)

S3 Table Models used to fit the data in the presence of cAMP. rX_n_, indicates corresponding models in S1 Table. For st, ze, f, sumz, p, nd p, *RSS*, MSE^*^ and CD see S1 Table. V in the models means that the step to the additional closed state C_1_^*^ was assumed to depend on voltage.
(DOCX)

S1 Fig Global fit of 27 activation and deactivation time courses according to a model proposed by Altomare and coworkers (model 14_n_,13_a_) [38]. Experimental traces and fitted curves are given in black and red color, respectively. Shades of gray indicate s.e.m. For further explanation see text. (**a**) Model scheme. (**b**) Fit of the *P*_o_ time courses in the absence of cAMP. (**c**) Fit of the *P*_o_ time courses in the presence of cAMP.
(TIF)

S2 Fig Global fit of the 27 activation and deactivation time courses in the presence of cAMP by model 1_n_. (**a**) Structure of model 1_n_. (**b**) Fit of the same traces as in S1C Fig. All rate constants at zero mV were free parameters. The gating charges *z*_1_ and *z*_2_ were fixed to the values provided for model 1_n_ in Table 1. Model 1_n_ with its gating charges is inadequate to describe the traces in the presence of cAMP.
(TIF)

S3 Fig Voltage dependencies of the effective rate constants in models 1_n_ and 1_a_. The rate constants were computed according to the data of Table 1 in combination with S2 Table. The rate constants of the voltage-dependent steps in the models are plotted in log-lin diagrams and these diagrams are superimposed to the respective transitions in the models.
(TIF)

S4 Fig Effect of cAMP on Boltzmann relationships at different pulse durations. The curves were computed with the models 1_n_ and 1_a_ for the absence and presence of 10 μM cAMP. Plotted is the open probability at the end of voltage pulses of either 1, 4 or 15 s duration as well as at true steady-state conditions.
(TIF)

S1 Data Current traces used for the calculations. (ZIP)
