# Supplementary material for: Activation gating in HCN2 channels
Source: PLoS Comput Biol. 2018 Mar 22;14(3):e1006045. doi: 10.1371/journal.pcbi.1006045 (PMC5863937; doi:10.1371/journal.pcbi.1006045)
Supplement: S1 Text — (DOCX) [file pcbi.1006045.s001.docx]

**Supplementary Methods**

**Electrophysiology**

Currents were recorded with the patch-clamp technique from inside-out macropatches obtained from the oocytes expressing mHCN2 channels. The patches contained between ~200 and ~4000 channels. The patch pipettes were pulled (P-2000, Sutter Instrument, Novato (CA), USA) from quartz tubing (VitroCom, New Jersey, USA) with an outer and inner diameter of 1.0 and 0.7 mm with a resistance of 1.0-1.9 MΩ. The bath solution contained (in mM) 100 KCl, 10 EGTA, 10 Hepes, (pH 7.2) and the pipette solution contained (in mM) 120 KCl, 10 Hepes, 1.0 CaCl_2_ (pH 7.2). To study the voltage-dependent gating at saturating cAMP, 10 μM cAMP (Sigma-Aldrich Corp., St. Louis, USA) was added to the bath solution.

Currents were recorded at room temperature (18-20°C) with an Axopatch 200B amplifier (Axon Instruments Inc., Foster City (CA), USA). To minimize the run down typical for HCN channels in inside-out patches [1, 2] recording was started 210 seconds after the formation of the inside-out patch. The holding potential was generally 0 mV and the interval between two double pulses was 8 seconds. Stimulation and data recording were performed with the ISO3 hard- and software (16-bit resolution, MFK, Niedernhausen, Germany) and with Patchmaster and LIH 8+8 hard- and software (HEKA Elektronik Dr. Schulze GmbH, Lambrecht, Germany). The sampling rate was 1 kHz and the on-line filter was set to 5 kHz (4-pole Bessel). The pulse protocols are described together with the data.

**Computation of Boltzmann functions**

Steady-state activation relationships in Fig. 1b were computed with the Boltzmann equation according to

*I*/*I* _maxcAMP,satV_ = *I*_satV_/*I*_maxcAMP,satV_ (1+exp[*zF*(*V*-*V*_h_)/*RT*])^-1^. (S1)

*I* is the current amplitude at actual voltage and actual cAMP, *I*_satV_ is the current amplitude at saturating hyperpolarizing voltage and actual cAMP, and *I*_maxcAMP,satV_ is the current amplitude at saturating hyperpolarizing voltage and saturating cAMP (10 µM). *z* is the gating charge, *F* the Faraday constant, *R* the molar gas constant, *T* the temperature in K and *V*_h_ the voltage of half maximum activation.

**Transformation of current traces into open probability time courses**

Each applied pulse protocol consisted of a sequence of a hyperpolarizing and depolarizing pulse to record activation and deactivation time courses, respectively. Statistical outlier points, appearing occasionally at strong hyperpolarization, were eliminated from the traces by an interpolation routine using either a polynomial or exponential. The traces were corrected for linear leak and capacitive current components using appropriate pulses from 0 to +50 mV.

The currents in the absence of cAMP were transformed into time courses of the open probability (*P*_o_) as follows: First the current deactivation time courses were scaled to the amplitude of the respective current activation time courses by setting the mean current amplitude in the intervals 5-10, 5-15 and 5-20 ms to the late current amplitude of the respective activation time course. Then the current amplitude after 4 s activation was set to the *P*_o_ value according to the *P*_o_-V relationship of a previous report [3], yielding the values 0.711±0.029, 0.572±0.028 and 0.11±0.018 at -140mV, -125 mV, -110 mV, respectively. The errors were treated according to the error propagation law and they are shown in shades of gray in Fig. 2b, d. Shorter activation time courses were normalized according to the mean of the respective longer activations at the corresponding time point.

For the current time courses in the presence of 10 µM cAMP, it was assumed that after 3 s at -130 mV a steady-state value of 0.99±0.01 was reached [4]. All other open probabilities were related to this maximal value. The transformation of the current time courses into *P*_o_ time courses was performed as described above. The reversal potential was generally assumed to be +4.6 mV [3].

**Calculation of voltage-dependent rate constants and model assumptions**

Voltage-dependent rate constants for the forward (*k*_f_) and backward (*k*_b_) reaction were described according to

*k*_f_ = *k*_f_^0^ exp(-0.5×*z_x_VF*/*RT*) and (S2a)

*k*_b_ = *k*_b_^0^ exp(0.5×*z_x_VF*/*RT*), (S2b)

respectively. Here *k*_f_^0^ and *k*_b_^0^ denote a rate constant at zero mV in s^-1^, *z*_x_ is the number of gating charges in step *x*.

The following assumptions were adopted:

1. The gating charge *z*_x_ was assumed to be equal in corresponding steps of the closed and open channel.
2. The energy barrier was assumed to be symmetric.
3. The condition of detailed balance was assumed.

The open probability *P*_o_ at maximum voltage activation upon zero or saturating cAMP, 0.71 and 0.99, respectively, were generally used to specify the respective closed-open isomerization apart from the Altomare model (c.f. S1 Fig).

**Global fit strategies**

For a Markov model with given rate constants, time courses following voltage pulse protocols were calculated with the help of the master equation as described by van Kampen [5]. For each model, the rate constants were determined by globally fitting all *n*_t_=27 averaged time courses of *P*_o_. For each activation and deactivation interval 30 fit points were used, resulting in *n*_d_=60 fit points per trace. Fit results were obtained by minimizing the residual sum of squares (*RSS*) at the fit points by a Levenberg-Marquardt algorithm [6] according to

$$RSS=\sum_{k=1}^{nt*nd} \left( yDk-yMk \right)2.$$

Here, *y*_D_*_k_* and *y*_M_*_k_* are the *P*_o_ of the data and model, respectively. The minimized *RSS* is an absolute measure for the goodness of the fit, without considering the number of parameters *n*_p_. To determine standard errors of the fitted parameter values, the mean square error (*MSE*) was calculated according to

(S3)

(S4)

$$MSE = \frac{RSS}{nt*nd- np}.$$

With the help of the *i*-th entry of the covariance matrix main diagonal (*cov_ii_*) the standard error for the *i*-th parameter *se(p_i_)* was calculated as

$se\left( p_{i} \right)= \sqrt{MSE*{cov}_{ii}}.$ (S5)

To get a measure for the goodness of a fitted model independent of the number of fit points also sufficiently penalized by an increased number of parameters, *RSS* was normalized with respect to the number of fit points per trace according to

(S6)

$${RSS}^{*}=\frac{1}{nd}\sum_{k=1}^{nt*nd} \left( yDk-yMk \right)2.$$

Now each data trace provides only one degree of freedom. This results in a normalized *MSE* value given by

(S7)

$${MSE}^{*}= \frac{{RSS}^{*}}{nt-np} ,$$

which was used for the model ranking. Respective fit strategies were used in [7, 8].

In case of the fits with models containing an additional slow transition to a closed state C^*^ outside the activation pathway (model 1_a_), a further constraint had to be introduced into the fit to realize that after an interval of 7 s at 0 mV deactivation was completed, as observed in the experiments. Practically this was realized by artificially concatenating the activation time course at -130 mV after a recovery interval of 7 s at 0 mV to the actual time course and fitting this time course together with the time course. However, for the model ranking, the *MSE*^*^ values and the parameter errors were calculated for the traces without these time courses to reach comparability. It should be emphasized that the wide voltage range of more than 200 mV covered by our global analyses caused that each voltage-dependent rate constant reached at one of the extreme voltages values in the order of 10 s^-1^ or slower. This suggests that all rate constants are determined by our data.

**Probability flux densities**

The time courses of the probability *P*_X_(*t*) that an individual state X is occupied during the activation and deactivation gating were computed using the rate constants obtained by the global fits. From *P*_X_(*t*) the time courses of the unidirectional probability flux density from state X to an adjacent state Y were obtained by

*f*_u,XY_(*t*) = *P*_X_(*t*) *k*_XY_, (S8)

where *k*_XY_ is the rate constant determining the transition from state X to state Y. The net probability flux density between two adjacent states was obtained as difference between the forward and backward unidirectional flux density for two adjacent states according to

*f*_XY_ = *f*_u,XY_ - *f*_u,YX_ . (S9)

A flux in the direction to O_2_ was attributed a positive sign.

**Numerical Calculations**

All calculations were performed with Matlab R2012b. We developed own program tools and a graphical user interface to implement the described fit routines.

**References**

1. DiFrancesco, D., A. Ferroni, M. Mazzanti, and C. Tromba. 1986. Properties of the hyperpolarizing-activated current (if) in cells isolated from the rabbit sino-atrial node. The Journal of physiology 377:61-88.

2. Pian, P., A. Bucchi, R. B. Robinson, and S. A. Siegelbaum. 2006. Regulation of gating and rundown of HCN hyperpolarization-activated channels by exogenous and endogenous PIP2. J Gen Physiol 128:593-604.

3. Kusch, J., C. Biskup, S. Thon, E. Schulz, V. Nache, T. Zimmer, F. Schwede, and K. Benndorf. 2010. Interdependence of receptor activation and ligand binding in HCN2 pacemaker channels. Neuron 67:75-85.

4. Thon, S., R. Schmauder, and K. Benndorf. 2013. Elementary functional properties of single HCN2 channels. Biophys J 105:1581-1589.

5. Van Kampen, N. G. 2007. Stochastic processes in physics and chemistry. Elsevier.

6. Brown, K. M., and J. E. Dennis. 1972. Derivative-free analogues of the Levenberg-Marquardt and Gauss algorithmus or nonlinear least squares approximation. Num. Math. 18:289-297.

7. Kusch, J., S. Thon, E. Schulz, C. Biskup, V. Nache, T. Zimmer, R. Seifert, F. Schwede, and K. Benndorf. 2012. How subunits cooperate in cAMP-induced activation of homotetrameric HCN2 channels. Nat Chem Biol 8:162-169.

8. Nache, V., T. Eick, E. Schulz, R. Schmauder, and K. Benndorf. 2013. Hysteresis of ligand binding in CNGA2 ion channels. Nature communications 4:2864.
