# Supplementary material for: Activation gating in HCN2 channels
Source: PLoS Comput Biol. 2018 Mar 22;14(3):e1006045. doi: 10.1371/journal.pcbi.1006045 (PMC5863937; doi:10.1371/journal.pcbi.1006045)
Supplement: S1 Table — Cx and Ox mean closed and open states respectively. In coupled-dimer models the allosteric step leads to the flipped state Fx. Opening appears only if both dimers are in a flipped state. This is indicated by the index CD→O. st, stoichiometric factors used; ze, gating charge z equal in all steps, f, allosteric factor used; sumz, sum of gating charges; p, number of parameters; nd p, number of parameters not determined given by a standard error >60%, RSS, residual sum of squares of all 27 traces; MSE*, normalized mean square error given by equation S7; rXa, indicates corresponding models in S3 Table. CD means coupled dimer. (DOCX) [file pcbi.1006045.s002.docx]

| No. | Scheme  C_1_  C_0_  C_2_  O_0_  O_1_  O_2_ | st | ze | f | sumz | p | nd p | RSS | MSE* | r X_a_ |
| --- | --- | --- | --- | --- | --- | --- | --- | --- | --- | --- |
| 1_n_ | C_1_  C_0_  F_0_  F_1_  F_2_  C_2_  CD🡪O |  |  |  | 8.05 | 12 | 2 | 1.81E-01 | 2.02E-04 | 5_a_ |
| 2_n_ |  |  |  |  | 6.81 | 12 | 0 | 2.20E-01 | 2.44E-04 |  |
| 3_n_ | C_1_  C_0_  C_2_  C_3_  O_0_  O_1_  O_2_  O_3_  C_1_  C_0_  C_2_  C_3_  O_0_  O_1_  O_2_  O_3_  C_4_  O_4_ |  |  |  | 15.05 | 17 | 7 | 1.52E-01 | 2.53E-04 | 6_a_ |
| 4_n_ |  |  |  |  | 21.53 | 22 | 16 | 1.38E-01 | 4.59E-04 |  |
| 5_n_  C_1_  C_0_  C_2_  C_3_  O_0_  O_1_  O_2_  O_3_  C_4_  O_4_  C_1_  C_0_  C_2_  O_0_  O_1_  O_2_ |  | x |  |  | 10.06 | 10 | 1 | 5.31E-01 | 5.21E-04 |  |
| 6_n_ |  | x |  |  | 7.82 | 8 | 2 | 6.32E-01 | 5.55E-04 |  |
| 7_n_ | CD🡪O  C_1_  C_0_  C_2_  F_0_  F_1_  F_2_ | x |  |  | 5.36 | 8 | 0 | 7.25E-01 | 6.36E-04 |  |
| 8_n_ | C_1_  C_0_  C_2_  C_3_  O_0_  O_1_  O_2_  O_3_ | x |  |  | 7.97 | 9 | 1 | 7.22E-01 | 6.68E-04 |  |
| 9_n_ | C_1_  C_0_  C_2_  O_0_  O_1_  O_2_ |  | x |  | 6.24 | 11 | 7 | 6.56E-01 | 6.83E-04 | 11_a_ |
| 10_n_ | C_1_  C_0_  O_0_  O_1_ |  |  |  | 6.44 | 7 | 2 | 8.66E-01 | 7.21E-04 |  |
| 11_n_ | C_1_  C_0_  C_2_  O_0_  O_1_  O_2_ | x | x |  | 9.75 | 7 | 2 | 8.91E-01 | 7.42E-04 |  |
| 12_n_ | C_1_  C_0_  C_2_  C_3_  O_0_  O_1_  O_2_  O_3_ | x | x |  | 12.62 | 7 | 0 | 9.26E-01 | 7.72E-04 |  |
| 13_n_ | C_1_  C_0_  C_2_  C_3_  O_0_  O_1_  O_2_  O_3_  C_4_  O_4_  C_1_  C_0_  C_2_  C_3_  O_0_  O_1_  O_2_  O_3_  C_4_  O_4_  ALTOMARE | x | x |  | 11.36 | 7 | 0 | 9.43E-01 | 7.86E-04 |  |
| 14_n_ |  | x | x | x | 10.55 | 7 | 0 | 1.07E+00 | 8.89E-04 |  |
