# Supplementary material for: Activation gating in HCN2 channels
Source: PLoS Comput Biol. 2018 Mar 22;14(3):e1006045. doi: 10.1371/journal.pcbi.1006045 (PMC5863937; doi:10.1371/journal.pcbi.1006045)
Supplement: S2 Table — From the fit parameters (Table 1) the rates (s-1) were determined as listed. Po,sat was set to 0.71 and 0.99 in the absence and presence of cAMP, respectively [47, 52]. *indicates rate constants used only in model 1a. (DOCX) [file pcbi.1006045.s003.docx]

| rate constant | calculation |
| --- | --- |
| *k*_1_ | *k*_1_^0^ exp[-0.5×*z*_1_*VF*/*RT*] |
| *k*_2_ | *k*_2_^0^ exp[0.5×*z*_1_*VF*/*RT*] |
| *k*_3_ | *k*_3_^0^ exp[-0.5×*z*_2_*VF*/*RT*] |
| *k*_4_ | *k*_4_^0^ exp[0.5×*z*_2_*VF*/*RT*] |
| *k*_5_ | *k*_5_ |
| *k*_6_ | microscopic reversibility |
| *k*_7_ | *k*_7_ |
| *k*_8_ | *k*_7_ (1-*P*_o,sat_)/*P*_o,sat_ |
| *k*_9_ | *k*_9_^0^ exp[-0.5×*z*_1_*VF*/*RT*] |
| *k*_10_ | *k*_10_^0^ exp[0.5×*z*_1_*VF*/*RT*] |
| *k*_11_ | *k*_11_^0^ exp[-0.5×*z*_2_*VF*/*RT*] |
| *k*_12_ | *k*_12_^0^ exp[0.5×*z*_2_*VF*/*RT*] |
| *k*_13_* | *k*_13_^0^exp[-0.5×*z*_c_*VF*/*RT*] |
| *k*_14_* | *k*_14_^0^exp[0.5×*z*_c_*VF*/*RT*] |
