# Supplementary material for: Activation gating in HCN2 channels
Source: PLoS Comput Biol. 2018 Mar 22;14(3):e1006045. doi: 10.1371/journal.pcbi.1006045 (PMC5863937; doi:10.1371/journal.pcbi.1006045)
Supplement: S3 Table — For st, ze, f, sumz, p, nd p, RSS, MSE* and CD see S1 Table. V in the models means that the step to the additional closed state C1* was assumed to depend on voltage. (DOCX) [file pcbi.1006045.s004.docx]

| No. | Scheme | st | ze | f | sumz | p | nd p | RSS | MSE* | r X_a_ |
| --- | --- | --- | --- | --- | --- | --- | --- | --- | --- | --- |
| 1_a_ | C_1_  C_0_  C_2_  O_0_  O_1_  O_2_  C_1_^*^  V |  |  |  | 5.12 | 15 | 0 | 3.01E-01 | 4.19E-04 |  |
| 2_a_  C_1_  C_0_  C_2_  C_3_  O_0_  O_1_  O_2_  O_3_  C_1_^*^  V | C_1_  C_0_  C_2_  F_0_  F_1_  F_2_  C_1_^*^  V  CD🡪O |  |  |  | 5.14 | 15 | 5 | 3.17E-01 | 4.41E-04 |  |
| 3_a_  C_1_  C_0_  C_2_  O_0_  O_1_  O_2_  C_1_^*^ |  |  |  |  | 5.52 | 20 | 4 | 1.87E-01 | 4.45E-04 |  |
| 4_a_ |  |  |  |  | 4.94 | 14 | 4 | 4.64E-01 | 5.94E-04 |  |
| 5_a_ | C_1_  C_0_  C_2_  O_0_  O_1_  O_2_ |  |  |  | 3.84 | 12 | 3 | 6.34E-01 | 7.05E-04 | 1_n_ |
| 6_a_ | C_1_  C_0_  C_2_  C_3_  O_0_  O_1_  O_2_  O_3_  C_1_  C_0_  C_2_  C_3_  O_0_  O_1_  O_2_  O_3_  C_4_  O_4_ |  |  |  | 9.06 | 17 | 6 | 4.51E-01 | 7.51E-04 | 3_n_ |
| 7_a_ |  | x | x | x | 6.39 | 5 | 0 | 1.01E+00 | 7.67E-04 |  |
| 8_a_ | C_1_  C_0_  C_2_  O_0_  O_1_  O_2_  C_1_^*^ | x |  |  | 4.72 | 11 | 0 | 8.08E-01 | 8.65E-04 |  |
| 9_a_  C_1_  C_0_  C_2_  C_3_  O_0_  O_1_  O_2_  O_3_  C_1_^*^ |  |  |  |  | 5.59 | 20 | 10 | 4.01E-01 | 9.56E-04 |  |
| 10_a_ | C_1_  C_0_  C_2_  C_3_  O_0_  O_1_  O_2_  O_3_  C_4_  O_4_ | x | x | x | 6.29 | 6 | 0 | 1.29E+00 | 1.02E-03 |  |
| 11_a_ | C_1_  C_0_  C_2_  O_0_  O_1_  O_2_ |  | x |  | 3.90 | 11 | 5 | 1.04E+00 | 1.08E-03 | 9_n_ |
| 12_a_ | C_1_  C_0_  C_2_  O_0_  O_1_  O_2_  C_1_^*^ | x | x |  | 4.44 | 10 | 2 | 1.15E+00 | 1.13E-03 |  |
| 13_a_ | C_1_  C_0_  C_2_  C_3_  O_0_  O_1_  O_2_  O_3_  C_4_  O_4_  ALTOMARE | x | x | x | 6.43 | 7 | 0 | 1.37E+00 | 1.14E-03 |  |
