# Supplementary figures and images for: Activation gating in HCN2 channels
Source: PLoS Comput Biol. 2018 Mar 22;14(3):e1006045. doi: 10.1371/journal.pcbi.1006045 (PMC5863937; doi:10.1371/journal.pcbi.1006045)

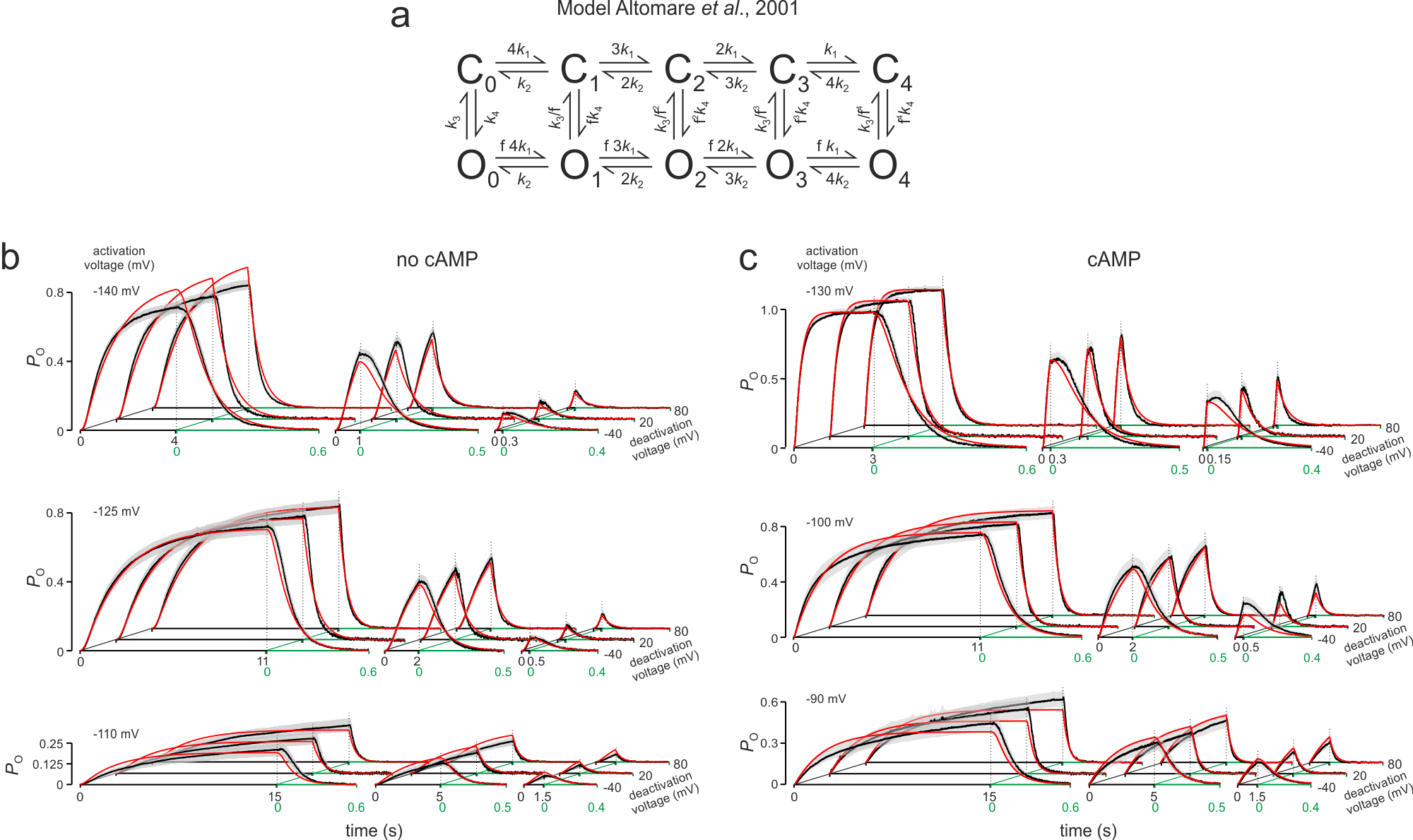

Supplement: S1 Fig — Experimental traces and fitted curves are given in black and red color, respectively. Shades of gray indicate s.e.m. For further explanation see text. (a) Model scheme. (b) Fit of the Po time courses in the absence of cAMP. (c) Fit of the Po time courses in the presence of cAMP. (TIF) [file pcbi.1006045.s005.tif]

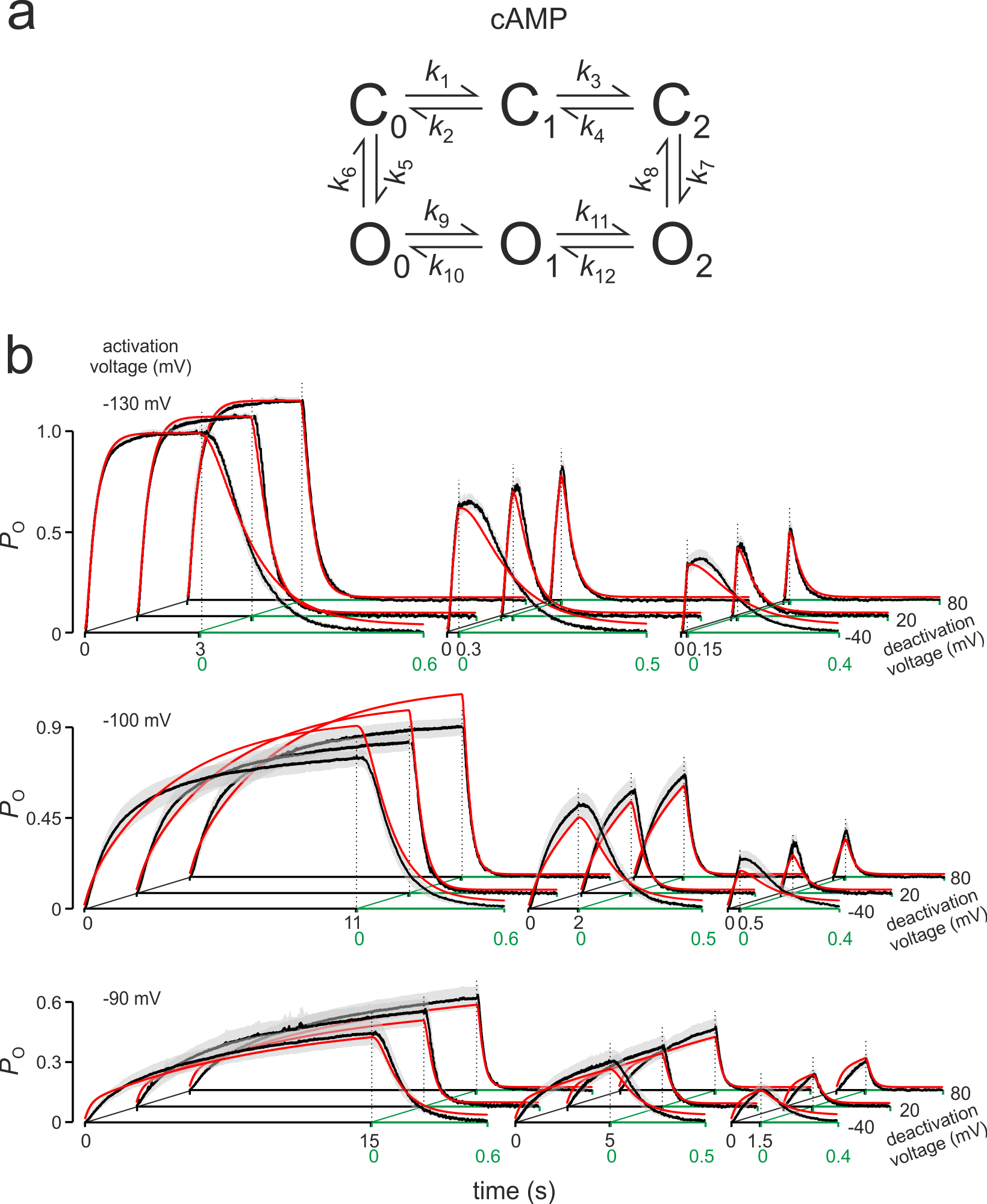

Supplement: S2 Fig — (a) Structure of model 1n. (b) Fit of the same traces as in S1C Fig. All rate constants at zero mV were free parameters. The gating charges z1 and z2 were fixed to the values provided for model 1n in Table 1. Model 1n with its gating charges is inadequate to describe the traces in the presence of cAMP. (TIF) [file pcbi.1006045.s006.tif]

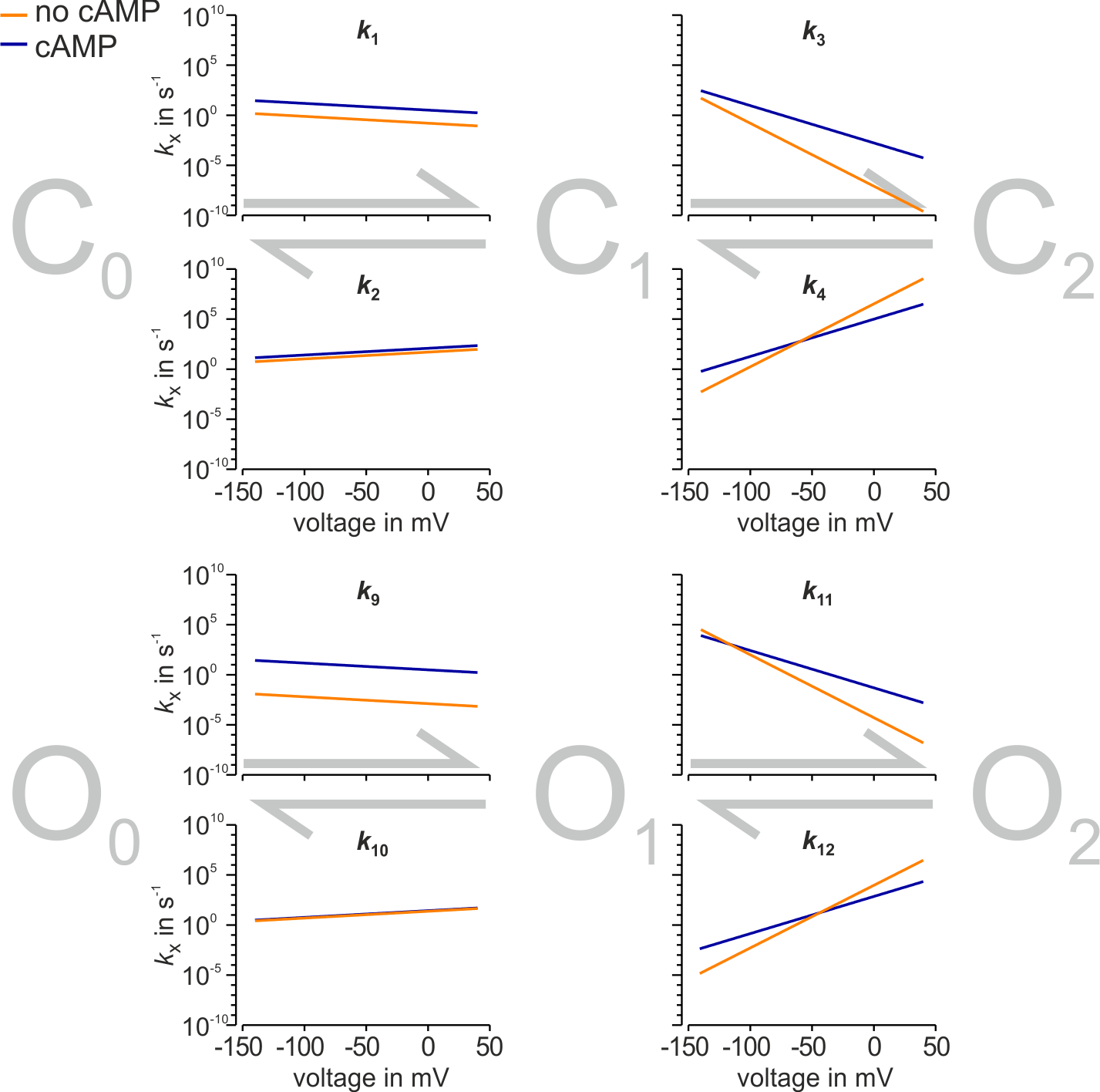

Supplement: S3 Fig — The rate constants were computed according to the data of Table 1 in combination with S2 Table. The rate constants of the voltage-dependent steps in the models are plotted in log-lin diagrams and these diagrams are superimposed to the respective transitions in the models. (TIF) [file pcbi.1006045.s007.tif]

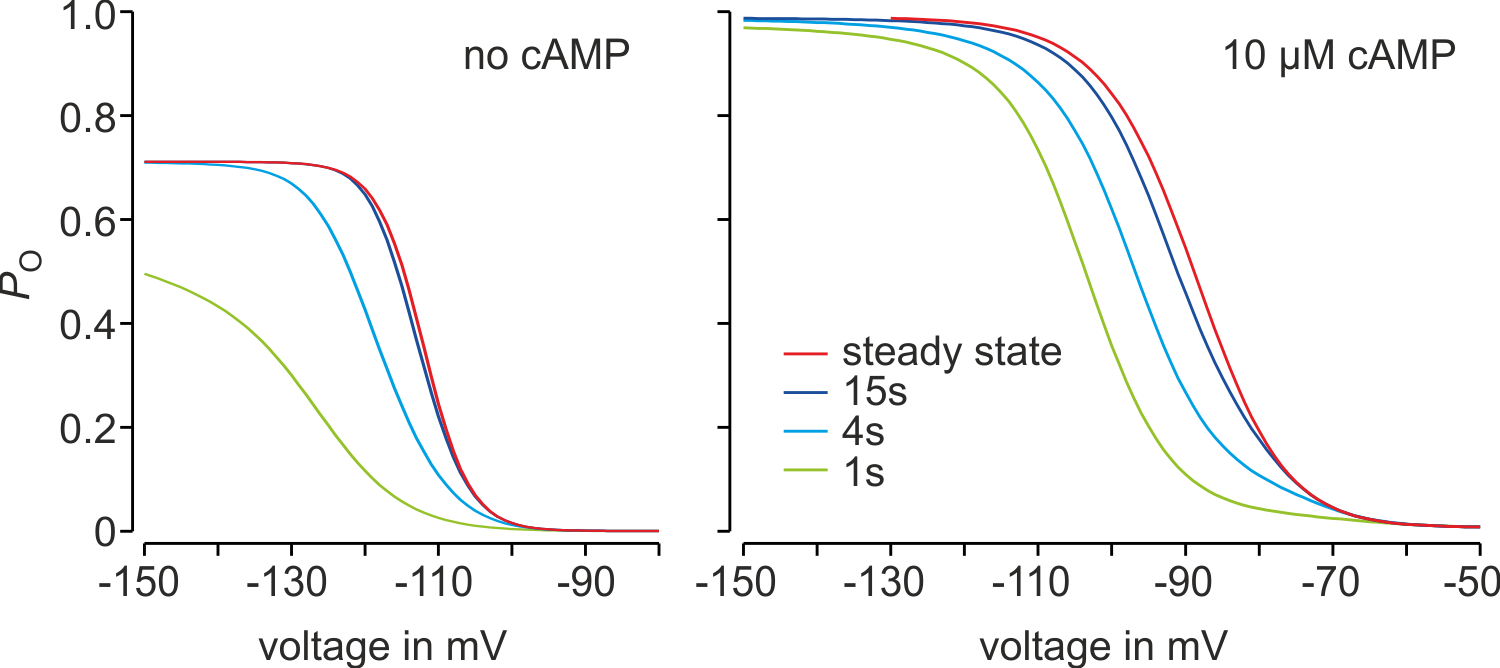

Supplement: S4 Fig — The curves were computed with the models 1n and 1a for the absence and presence of 10 μM cAMP. Plotted is the open probability at the end of voltage pulses of either 1, 4 or 15 s duration as well as at true steady-state conditions. (TIF) [file pcbi.1006045.s008.tif]
